# A Robust Document Identification Framework through f-BP Fingerprint

**DOI:** 10.3390/jimaging7080126

**Published:** 2021-07-29

**Authors:** Francesco Guarnera, Oliver Giudice, Dario Allegra, Filippo Stanco, Sebastiano Battiato, Salvatore Livatino, Vito Matranga, Angelo Salici

**Affiliations:** 1 Department of Mathematics and Computer Science, University of Catania, 95125 Catania, Italy; giudice@dmi.unict.it (O.G.); allegra@dmi.unict.it (D.A.); filippo.stanco@unict.it (F.S.); battiato@dmi.unict.it (S.B.); 2 School of Physics, Engineering and Computer Science, University of Hertfordshire, Hatfield AL10 9AB, UK; s.livatino@herts.ac.uk; 3 Raggruppamento Carabinieri Investigazioni Scientifiche, RIS di Messina, 98122 Messina, Italy; vito.matranga@carabinieri.it (V.M.); angelo.salici@carabinieri.it (A.S.)

**Keywords:** document identification, binary pattern, texture fingerprint

## Abstract

The identification of printed materials is a critical and challenging issue for security purposes, especially when it comes to documents such as banknotes, tickets, or rare collectable cards: eligible targets for ad hoc forgery. State-of-the-art methods require expensive and specific industrial equipment, while a low-cost, fast, and reliable solution for document identification is increasingly needed in many contexts. This paper presents a method to generate a robust fingerprint, by the extraction of translucent patterns from paper sheets, and exploiting the peculiarities of binary pattern descriptors. A final descriptor is generated by employing a block-based solution followed by principal component analysis (PCA), to reduce the overall data to be processed. To validate the robustness of the proposed method, a novel dataset was created and recognition tests were performed under both ideal and noisy conditions.

## 1. Introduction and Related Works

The manufacturing process needed to produce common paper sheets involves the use of wood particles with subsequent application of other compounds. The intrinsic random imperfections generated make the sheet almost unique, and under certain conditions it is possible to extract a proper fingerprint. The massive demand of robust identification methods in many contexts [1,2,3,4,5,6], makes fingerprint extraction from a sheet of paper an attractive and challenging research topic. Investigative scenarios in the forensic field [7,8], could gain several advantages from the availability of such a fingerprint.

Although several techniques have been proposed, most of them require expensive industrial devices [9,10], which are not commonly affordable. Taking inspiration from the use of wood fiber patterns for fingerprint extraction [11], the main objective of this work is the design of a cheaper solution able to extract a robust fingerprint. Please note that in contrast with a biometric system [12] the main interest is in finding a robust strategy to recognize a specific paper sheet by matching it with a previously acquired version of itself. Local Binary Pattern (LBP) [13] and its variants [14] have been employed under different conditions and global experimental settings, clearly outperforming the results obtained by Guarnera et al. [15] in terms of efficiency and effectiveness. It is worth noting that since in ideal conditions the paper texture is unique, any sufficiently descriptive image-processing approach should perform with good results in terms of accuracy. This led us to further investigate the problem by performing tests on papers where some degradation was synthetically applied (e.g., stain, crop, etc.) simulating real case scenarios where part of the original information is totally or partially missing.

However, the identification of a document for legal purposes, employed to detect counterfeiting and piracy, is usually done through the use of different techniques [7]. The interest in anti-counterfeiting measures, based on the fingerprints left on the surface of the paper without any specific embedding requirement, is the core of the present study. Other techniques are based on security patterns or on properly generated features that are hidden in the substrate material or masked by special ink properties. Such identification strategies are widely used, but typically require an additional pattern to be added, and are also expensive and hard to generalize for all cases (e.g., legal documents, banknotes, etc.). By contrast, strategies that directly analyze the physical properties of the material by not adding any signal, as in the case of common active methods (e.g., watermarking) are highlighted in this paper. The underlying hypothesis for the development of a fingerprint extraction technique is the existence of low-cost physically unclonable functions (PUFs) to generate an intrinsic random physical feature for paper identification with the following two properties:

- Fast and deterministic processing to obtain a response;
- The return of a unique response for the same request.

The response must be unpredictable, even for an attacker with physical access to the object, by operating as a sort of random function. The paper surface presents an inherently unique structure, as it consists of overlapping and inter-twisted wood fibers. Hence, the imperfections of a paper sheet caused by the manufacturing process can be exploited to uniquely identify such sheet. The use of a fingerprinting technique for document identification was proposed for the first time by Buchanan et al. [16]. It has been proven that is extremely unlikely that two document surfaces created with the same raw materials will be identical, although they will present some similarities. This fingerprint makes forgery unfeasible, given that it is unique and virtually impossible to modify. To extract a fingerprint from the paper structure, the authors in [16] employed laser irradiation from four different angles and acquired the reflected energy. Inspired by Buchanan et al., the authors in [17] proposed an improvement based on correlation metrics between the acquired energy signals. Cowburn introduced the use of laser speckle for product identification ([18,19]). Clarkson et al. [20] proposed the extraction of 3D paper structure by scanning different orientations and employing a Voronoi distribution to build the fingerprint. Samsul et al. [9] proposed a fingerprint extraction method, which exploits CCD sensors and laser speckle, to employ the visible pattern of bright and dark spots generated by interference of two or more light beams with different phases. A similar approach has been proposed by Sharma et al. [10]. In contrast to [9], they employed a microscope to acquire the speckle pattern. In recent years, CNN-based methods have achieved great performance in image recognition and classification, but have high complexity and require GPUs to perform training.

The aforementioned approaches work well for paper fingerprint extraction, but they require industrial and specific equipment. Recently, this limitation was overcome by the works of Toreini et al. [11] and Wong et al. [21]. In [21] the authors proposed a strategy to extract paper surface imperfections by exploiting multiple shots taken by a mobile camera under semi-controlled light conditions; subsequently, they investigated selected candidates through ad hoc mathematical models for each camera-captured image [22]. Unlike previous works, Toreini et al. [11] did not detect surface imperfections, but captured the random arrangement of the wood fibers within the paper sheet. To extract the paper pattern, they exploited a consumer camera and a backlit surface. However, they printed a bounding box on the analyzed paper to simplify the automatic texture registration. Since in real scenarios this registration strategy is not applicable, a different acquisition framework is needed. Based on the same filter of [11], Chen et al. in [23] exploited the microscopic features of wood fibers to obtain similar patterns, using expensive equipment based on double cameras. As already demonstrated in [24], the random disposition of wood fibers on paper sheets makes possible the construction of a fingerprint virtually impossible to tamper with; hence, given the limits of the previous works in terms of costs, acquisition constraints, and robustness, in [15] the authors presented a novel fingerprint extraction strategy using specific low-cost image-acquisition equipment and a simpler and faster method based on local binary patterns. In this paper, further experiments on LBP variants are carried out, such as Local Ternary Pattern (LTP) [25], Statistical Binary Pattern (SBP) [26] and Complete modeling of Local Binary Pattern (CLBP) [27] to find the most descriptive binary pattern for fingerprint identification and tampering to achieve a more robust solution. In contrast to [9,10,16,17,18,19,20,23], the present work proposes a fingerprint extraction method that does not require expensive industrial equipment (e.g., laser, microscopes), but solely cheaper devices such as an RGB camera, as it is based on wood fiber translucent patterns. Additionally, the proposed approach overcomes a method based on translucent patterns such as the one by [11]; in fact, LBP descriptors used in [15] have already been proved to outperform Gabor filters employed in [11] in terms of effectiveness and efficiency. Since the intrinsic advantages of [15] over [11] were confirmed in previous research, we compare the proposed approach only with [15] and not with [11].

The main contributions of this paper can be summarized as follows:

1. A new fingerprint extraction method, based on LBP variants, which outperforms existing approaches in the field;
2. An optimization of a BP-based fingerprint that employs block subdivision and Principal Component Analysis (PCA);
3. A new public dataset that includes images acquired with both low-cost and high-end devices, showing wood fiber patterns, which is the one available to the best of our knowledge;

The remainder of this paper is organized as follows. The next section is devoted to briefly summarizing the state of the art in the field. Section 2 details the proposed fingerprinting extraction strategy; Section 3 describes the acquisition procedure of image data and the overall organization of the employed dataset; in Section 4, experimental results are reported together with a deep analysis on the results obtained by the proposed approach. Conclusions are reported in Section 6.

## 2. Fingerprint Extraction Process

**Figure 1:**
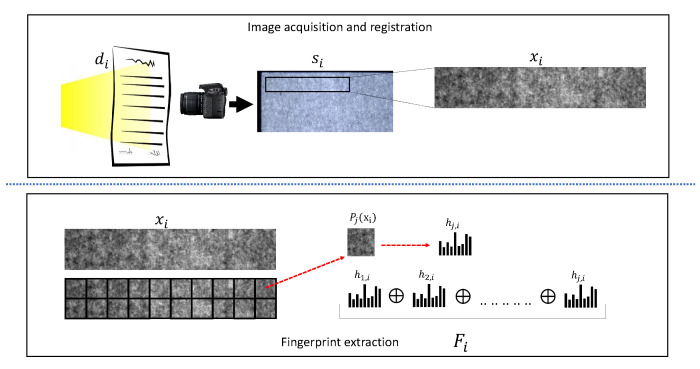
Overall pipeline of the proposed framework. First row describes the process to acquire documents; second row shows the fingerprint extraction process.

Illuminating the surface to highlight the wood fibers is mandatory to properly extract the pseudo-random pattern which is unique for each sheet of paper. However, such patterns must be digitalized and properly modeled mathematically to implement a robust document identification system, which is the goal of this paper. Given a certain physical paper document $d_{i}$, the aim of this work is to obtain a digital fingerprint $F_{i}$, namely a sequence of *K* ordered values $\left\{f_{i}^{\left(1\right)},f_{i}^{\left(2\right)},...,f_{i}^{\left(K\right)}\right\}$, which is solely determined by correctly processing the digital image $s_{i}$, which is the acquisition of the document $d_{i}$. The overall proposed pipeline is summarized in Figure 1.

### 2.1. Document Digitization and Image Registration Considerations

The physical set of *N* documents $D=\{d_{1},d_{2},...d_{N}\}$ was acquired using devices that are able to capture the wood fiber pattern by exploiting the translucent properties of the paper. In this work, two different acquisition environments were employed to compare the performance of low-end and high-end equipment. Details about devices and related settings are provided in Section 3. The acquisition of a physical document $d_{i}$ was carried out in a semi-constrained environment; specifically, the documents must be roughly aligned regarding the capturing device to guarantee an effective consequent registration. For the sake of readability, the set of the digitized versions of the documents $D=\{d_{1},d_{2},...d_{N}\}$ can be defined as $S=\{s_{1},s_{2},...s_{N}\}$.

To successfully analyze the wood fiber pattern of a document $d_{i}$, the related digital image $s_{i}$ must be registered. This step is critical as the paper fingerprint strongly depends on spatial information; hence, one must ensure that if a given document is acquired multiple times under the same setup, the system will process exactly the same region of the paper surface. To this aim, reference points were exploited (e.g., black bands in the acquired image) to rotate and properly crop $s_{i}$ (see Section 2.2 for more details). After registration, a W×H sample from each document $s_{i}$ was obtained, defined ad $x_{i}$, and the related set $X=\{x_{1},x_{2},...,x_{N}\}$ was employed to build the fingerprint.

### 2.2. Extracting a Unique Fingerprint

The extraction of a unique fingerprint from a sample $x_{i}$ is the process that encodes the texture information in such a way as to satisfy the following properties: (i) low complexity; (ii) encoding capabilities; (iii) robustness with respect to the missing parts. To this aim, the LBP descriptor and its variants [14] are employed, which are demonstrated to satisfy all the aforementioned requirements. These descriptors guarantee high capabilities in terms of discriminative power by maintaining low computational complexity and working almost perfectly even in the presence of slight variations on textures. In particular, LBP is a local descriptor that compares a pixel, called a pivot, to its *n* neighbors along the circle defined by a certain radius *r* [13]. In recent years the use of LBP for texture classification has grown, and a wide set of LBP variants has been proposed [14]. Hence, the so-called *f*-BP variant has the goal to improve the accuracy and the robustness for a specific task. The well-known local property makes the *f*-BP a flexible descriptor even in the presence of small perturbations, which is the fundamental requirement of the fingerprint we are looking for. Regardless of the *f*-BP, after pattern extractions the final descriptor is obtained by counting the times each pattern occurs, namely by computing a histogram.

Histograms are compact and effective descriptors for a various number of tasks; nevertheless, they heavily discard spatial information. To face this issue, $x_{i}$ is first divided in *M* non-overlapping p×p patches and the histogram is separately calculated for each patch $P_{j}\left(x_{i}\right)$ with j={1,2,.....,M}; hence the histogram $h_{i,j}$ represents the histogram of the *j* patch of the sample $x_{i}$. The importance of spatiality is easily guessed: if the document presents some types of fault (e.g., missing parts, tears, holes, noise) it is important they do not affect the whole fingerprint, but just a portion. For this reason, the choice of the patch size *p* and the hyperparameters $\theta_{f}$ of the employed *f*-BP variant (e.g., the number of neighbors *n* and the radius *r*) have consequences on the performance. The size *T* of the histogram depends on the number of possible patterns the *f*-BP variant led. For example, if one employs classical LBP with n=8 and r=1 the number of possible patterns, and the histogram size *T*, is 256. As far as the patch size *p* is concerned, large patches decrease spatial information while small patches make the BP excessively local and increase the complexity of the obtained fingerprint.

The final fingerprint $F_{i}$ for document $d_{i}$ can be obtained by concatenating all the histograms $h_{j,i}$ for j=1,2,...,M:

(1) $$F_{i}=\overset{M}{\underset{j=1}{⨁}}h_{j,i}$$

The size *K* of $F_{i}$ is K=M×T, as *M* patches are obtained from *M* histograms of size *T*. The goal of this study is to test different *f*-BP variants and look for the parameters $\left\{W,H,p,\theta_{f}\right\}$ which led to the most robust fingerprint.

## 3. Datasets for Document Identification and Fingerprint Testing

To evaluate the proposed approach and provide a great contribution to this research field, a new dataset is introduced, which is composed by 200 A4 paper sheets arranged in groups of 40 and divided into 5 non-overlapping classes. Each class is defined by two attributes: the manufacturer of the paper $b\in \{b_{1},b_{2},b_{3},b_{4}\}$ and the weight or grammage (measured in g/m^2^) g∈{80,160,200}. Thus, the obtained classes are the following: $\left(b_{1},80\right)$, $\left(b_{2},80\right)$, $\left(b_{3},80\right)$, (b4,160), (b4,200).

**Figure 2:**
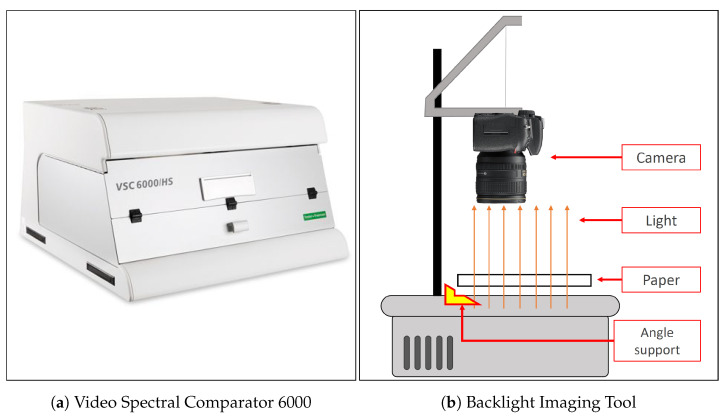
Devices employed for acquisitions.

All the 200 documents in D were then acquired multiple times using two different devices as described in Figure 2 and detailed in the next subsections. The dataset will be made available online after this paper is accepted and a download link will be placed in this section.

### 3.1. Devices

To compare the performances obtainable with high-end and low-end equipment, each document is digitized using two different devices. For the high-end case the Video Spectral Comparator 6000 (VSC) was employed while for the low-end one we used the Backlight Imaging Tool (BIT): a cheap overhead projector combined with a digital camera that we accurately designed.

The VSC consists of a main unit (Figure 2b) connected to a standard workstation. It provides several functionalities and a set of different light sources to highlight paper details normally not visible in standard conditions. Table 1 shows VSC acquisition settings.

The BIT consists of an overhead projector which serves as source light and a consumer RGB camera hung on the projector arm. The employed camera is a Nikon D3300 equipped with a Nikon DX VR 15 mm–55 mm 1: 3.5–5.6 GII lens. Settings details are listed in Table 2.

### 3.2. Dataset Acquisition

For the sake of clarity, the terms $S_{VSC}$ and $S_{BIT}$ will be employed for referring to the digital acquisitions made by the VSC and the BIT respectively. The overall dataset acquisition pipeline is depicted in Figure 1. As expected, $S_{VSC}$ and $S_{BIT}$ show different contrast and sharpness.

**Figure 3:**
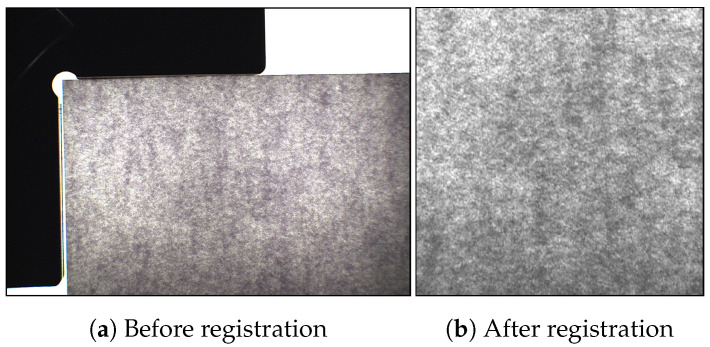
Document acquisition with VSC before registration (**a**) and after registration (**b**).

**Figure 4:**
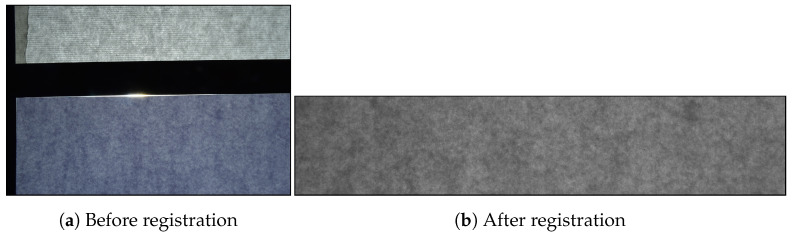
Document acquisition with BIT before (**a**) and after registration (**b**).

$S_{VSC}$ consists of 200 documents acquired twice, for a total of 400 acquisitions (Table 3). The result of a single acquisition is a bitmap image of 1292×978 pixels and 300 dot per inch (dpi), as reported in Figure 3a. $S_{BIT}$ consists of 200 documents acquired 8 times. However, the insufficient power of light in the BIT does not allow the extraction of the translucent pattern from paper with grammage 160 or 200. Thus, only the 120 documents with grammage 80 were considered for a total of 960 acquisitions with a resolution of 6000×4000 pixels and 300 dpi (Table 3). Figure 4a shows a raw acquisition, where the black bands, used for image registration, are visible.

### 3.3. Image Registration

The acquisition of the black bands outside the paper area surface was voluntarily performed to distinguish selectively the pixels from the external area and easily obtain a registered set of images. All the raw images in $S_{VSC}$ and $S_{BIT}$ were converted into grayscale. First, a luminance threshold is used to find the top-left corner $\left(y_{0},y_{1}\right)$ of the sheet of paper. Secondly, the image anchored in position ($y_{0}+u$, $y_{1}+u$) is cropped, where *u* is the minimum offset to perform a cropping by excluding the external area. The value of *u* is variable: the larger the external area acquired is, the greater will be its value. Images acquired by means of the VSC are cropped into patches of 400×400, while the ones acquired with the BIT are cropped into patches of 5000×1000 pixels. Finally, one obtained $X_{VSC}$, the set of 400 registered samples from VSC and $X_{BIT}$, the set of 960 registered samples from BIT. Source examples are shown in Figure 3b and Figure 4b.

## 4. Experiments and Discussion

To evaluate the proposed fingerprint extraction approach in depth, analysis of the datasets described in Section 3 were performed in terms of recognition tests. Since each document was acquired multiple times (i.e., twice for the VSC and 8 times for the BIT), a fingerprint reference dataset was built to face the recognition task; such reference datasets consist of only one sample per document while the rest of the samples were used for querying it. A certain document *d* with extracted fingerprint $F_{a}$ will have a correct match with the closest element in the reference dataset $F_{b}$, if both $F_{a}$ and $F_{b}$ “belong” to the document *d*; in other words, a correct match occurs if $s_{a}$ and $s_{b}$ are different acquisitions of the same document. The recognition test performances are measured using the well-known accuracy metric defined as the rate of queries, which obtain a correct match. The adopted similarity measure for fingerprints was the Bhattacharyya distance [28], which is typically and effectively employed for problems where probability distribution must be compared. However, to better assess the effectiveness of the proposed fingerprint, four different recognition experiments are performed as detailed in the following. First, the original LBP was employed to compare the recognition accuracy on both datasets (VSC and BIT) obtaining the demonstration of device invariance. Given this result, a comparison was performed only on the BIT dataset employing LBP fingerprints computed as in [15] vs. the three other LBP variants, i.e., LTP [25], SBP [26] and CLBP [27]. Moreover, also the fingerprint robustness was investigated. To this aim, a challenging scenario was created where the query samples were intentionally altered by removing some pixels from the digital image to simulate physical damage of the paper (e.g., tears, holes). Finally, an optimization in terms of fingerprint dimensions was carried out and tested as well by exploiting principal component analysis (PCA) [29].

### 4.1. Dataset Comparison

To demonstrate the goodness of the LBP-based fingerprint extraction method, we started from the work of Guarnera et al. [15], our previous work, which represents the state of the art. Table 4 shows the overall accuracy obtained in the recognition tests performed on both datasets: 96.5% and 99.2% for VSC and BIT, respectively. Although samples from different datasets have different patch sizes, the best for both datasets was 100×100. This is a reasonable trade-off to preserve local spatial information. The accuracy on the BIT dataset is slightly higher than the accuracy obtained on the VSC. This demonstrates that the robustness of the fingerprint does not depend on the acquisition settings nor device.

### 4.2. Comparisons among LBP Variants

As introduced in Section 2, many LBP variants were proposed for texture analysis. Among them, LTP [25], SBP [26] and CLBP [27] were selected for the experiments described in this section. In the previous section, the independence of the proposed fingerprint from the acquisition device was demonstrated. Starting from this evidence, in the next experiments, only the BIT dataset will be employed given the higher number of available samples. The results in terms of accuracy are reported in Table 5 where CLBP and SBP show an improvement in terms of performance vs. LBP, by achieving an accuracy of 99.7% and 99.4%, respectively. It is worth noting that LBP is the employed method of [15] to extract the fingerprint, so the aforementioned results represent the overperformance with respect to the state of the art. As described in the literature, LTP tends to work better than LBP when the texture presents regions that are uniform (i.e., low variance). It is worth noting that the wood fiber patterns show a high variance, thus explaining the worse results of such descriptor. SBP, which is a generalization of the common binary pattern, as expected, obtains accuracy results of (99.4%) that are slightly better than LBP. Finally, the best performance was obtained by CLBP (99.7%) even if it delivers the largest fingerprint in terms of histogram dimensions (number of bins).

### 4.3. Tests on Noisy Environment: Synthetically Altered Documents Are Introduced

**Figure 5:**
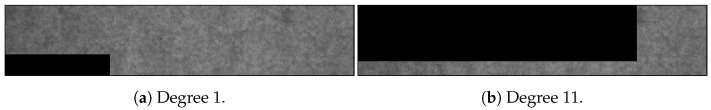
Examples of altered documents with simulations of tear damage; in particular (**a**) represents the first degree of damage while (**b**) the last (e.g., 11).

**Figure 6:**
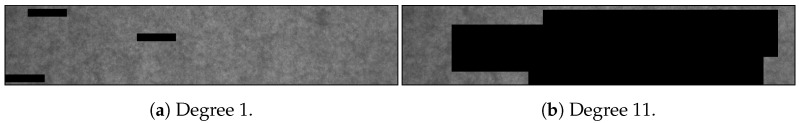
Examples of altered documents with simulations of stain damage; in particular (**a**) represents the first degree of damage while (**b**) the last (e.g., 11).

**Figure 7:**
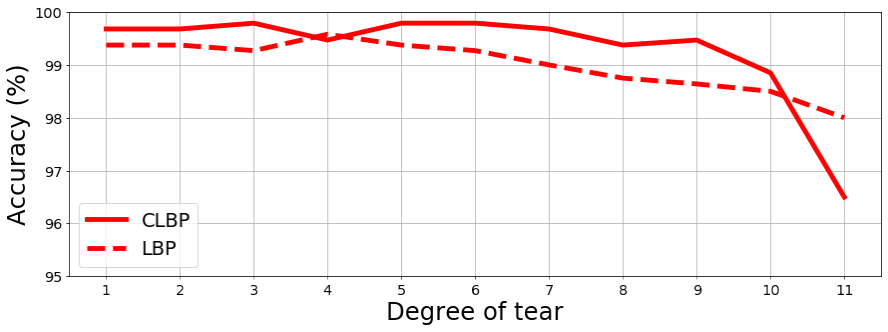
Accuracy employing CLB and LBP VS degrees of tear alteration.

**Figure 8:**
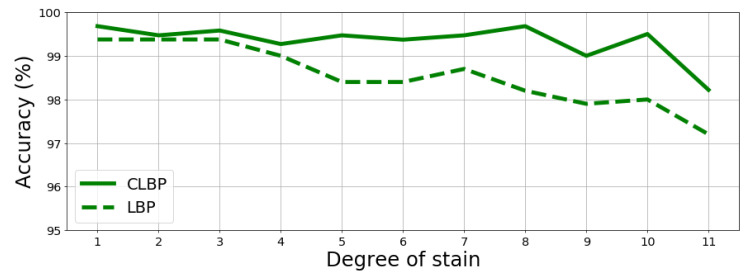
Accuracy of CLB and LBP VS degrees of stain alteration.

The proposed method for fingerprint extraction was tested under controlled conditions to properly assess what was expected to happen in real cases, namely when a document experienced some alteration between the first fingerprint extraction and the successive ones. Hence, the original fingerprint of the document may be very dissimilar from the latter one. To this aim, two types of damages on paper were simulated: tears and stain. The “tear” simulates a loss of information which starts from one angle of a sheet sample $x_{i}$ by replacing such loss with black pixels, while the “stain” introduces random black blocks on the sample to simulate holes or stains. For both, the so-called degree represents the size of black area: the maximum degree corresponds to about 75% of the full sample to be removed (see Figure 5 and Figure 6). Given the aforementioned alterations, a new recognition test on the BIT dataset was carried out, which includes 120 samples without any alterations on the fingerprint database and other 960 samples with alterations that were used to query the database. The results are reported in Figure 7 and Figure 8 further proving the robustness of the proposed fingerprint, specifically the CLBP-based one which achieves best performance once more.

### 4.4. Fingerprint Dimensions Optimization

All the tests described in the previous sections were performed employing the pipeline described in Figure 1 with the following settings: images were cropped into patches of 100×100 pixels; number of neighbors for CLBP were n=12 and radius was r=6. These settings brought to 500 patches from the BIT dataset; thus, a histogram of 8194 elements was computed for each patch. This results in a fingerprint with dimension of 500×8194= 4,097,000 elements whose storage occupancy is about 8.3 MB. Since the fingerprint with the proposed method could be even larger depending on parameters and since large fingerprints decrease efficiency, some optimization strategies to reduce it were explored.

The simplest strategy to reduce the fingerprint size was the increment of the patch size *p*; however, this could not guarantee the same accuracy performance. Table 6 shows the results obtained using larger values of *p* while monitoring the fingerprint size. The analysis of the results showed that the setting with p=200 presents a performance similar to p=100 (i.e., only a drop of 0.4% of accuracy) reducing the size by 25%, from 4,097,000 to 1,024,250 elements, that can be stored in 2.2 MB. However, as stated in the previous sections, employing bigger patches does not preserve spatial information and actually shows a tremendous accuracy drop (e.g., 67.6% for p=500).

**Figure 9:**
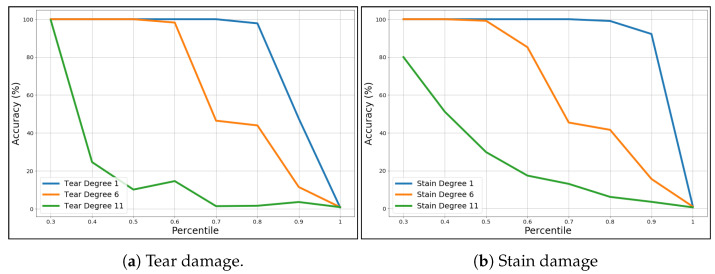
Accuracy variability of different percentiles on tear (**a**) and stain (**b**) damages.

To optimize the size of the fingerprint preventing a large loss in terms of accuracy, we employed the Principal Component Analysis (PCA) [29]. As we know, PCA reduces the dimensions by projecting each data point onto only some of the principal components to obtain lower-dimensional data while preserving most of the data variance; in a nutshell, it reduces the dimensions by preserving most of the information, which better describes a certain phenomenon. PCA is applied to each histogram $h_{j,i}$ previously obtained using CLPB. Hence, such histograms are drastically reduced in terms of dimensions. First, for testing purposes, all the 120 samples included in the fingerprint database are used to fit the PCA model. By employing the well-known explained variance analysis, we found that 95% of the information can be preserved using the first 32 principal components (also known as features), despite the original 8194. However, PCA moves histograms in a geometric space where the Bhattacharyya distance becomes less efficient; to face this problem, the recognition test was performed by means of the Euclidean distance. To verify the quality of reduction, the same recognition tests, as described in the previous sections, were carried out with the now-reduced fingerprints, delivering an accuracy of 97.97% with only 16,000 elements while maintaining the excellent performance of the not-reduced fingerprints case. It is worth noting that the PCA model was built using all the samples of each of the 120 documents in BIT. This could generate a PCA model overfitted on the data. Thus, a further test was performed using only the 50% of the dataset (60 documents) to fit the PCA model, while and we queried the reference dataset with the samples which come from the remaining 50%. In this case, it was found that 95% of information can be preserved using the first 40 principal components for each patch. recognition tests confirmed the results obtained with the PCA model built on all the 120 documents (97.97% of accuracy). It is important to note that although in the fingerprint comparison we also consider the missing parts when an alteration occurs, this does not heavily affect the Bhattacharyya distance between two fingerprints. On the contrary, the Euclidean distance is affected by this. In fact, the Euclidean distance calculated between an unaltered fingerprint of a document and an altered fingerprint of the same document exhibits extremely higher values, which impacts on the accuracy performance. To overcome this latter problem, a custom Euclidean distance was employed, where only a part of the fingerprint elements is considered in distance computation. Specifically, the differences between each element of the two fingerprints is computed and, subsequently, we sorted those differences by considering only a certain percentage of the lower ones. This percentage depends on the dimensions of the missing part, but this information is known by the operator during the identification phase, because in a real document the altered parts are visible. Figure 9a,b report the accuracy (vertical axis) when varying the percentage of elements included in distance computation (horizontal axis). The obtained results also suggest how to maintain a high accuracy according to the alteration degree. For example, in an average scenario of damage (orange lines) the 50% of distance is needed to maintain the accuracy over the 99%.

## 5. Fingerprint Robustness Analysis

The carried-out recognition tests started from the hypothesis that every query fingerprint $F_{q}$ could find a correspondent fingerprint $F_{x}$ into fingerprints database previously extracted from the same document and stored. A real case scenario could present some differences: the query fingerprint $F_{q}$ could not find a correspondent $F_{x}$ and then the nearest one has no meaning (it is the most similar but it is a fingerprint extracted from another document). Hence, additional information is needed: given the distance $\bar{e}$ between two samples. To solve this problem, $\bar{e}$ was analyzed in all the previously presented experiments; in particular, starting from the fingerprints extracted by the images acquired with BIT device (e.g., 960), the distances obtained in the tests without simulated damages employing CLBP and LBP were analyzed, considering three kinds of distances:

- ${\bar{e}}_{0}$: distance obtained between $F_{q}$ and $F_{x}$, both extracted from the same document, when $F_{x}$ is the closest fingerprint in the recognition test.
- ${\bar{e}}_{1}$: distance obtained between $F_{q}$ and $F_{x}$, both extracted from the same document, when $F_{x}$ is not the closest fingerprint in the recognition test.
- ${\bar{e}}_{null}$: distance obtained between $F_{q}$ and $F_{x}$, extracted from different documents.

Given 840 different $F_{q}$, 120 distances have been computed for each of them. For every $F_{q}$ analyzed, two results were obtained:

- The closest fingerprint $F_{x}$ is extracted from the same document of $F_{q}$, and then the distance between them is classified as ${\bar{e}}_{0}$ and the others 119 distances are classified as ${\bar{e}}_{null}$.
- The closest fingerprint $F_{x}$ is not extracted from the same document of $F_{q}$, and then the distance between them is classified as ${\bar{e}}_{1}$ and the others 119 distances are classified as ${\bar{e}}_{null}$.

It is easy to figure out that the population of ${\bar{e}}_{null}$ is much bigger than ${\bar{e}}_{0}$ and ${\bar{e}}_{1}$, whose sum is exactly 840.

**Figure 10:**
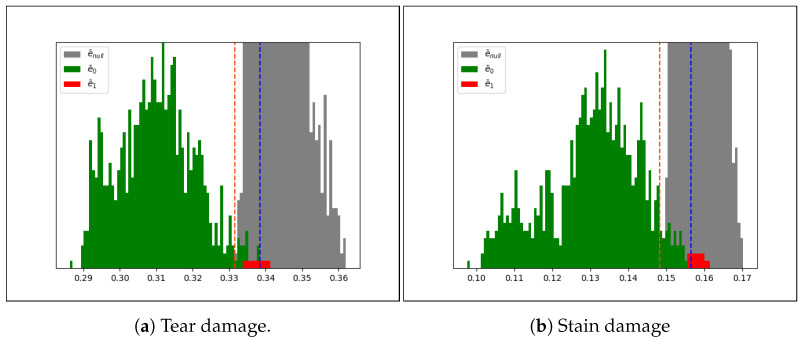
Accuracy variability of different percentiles on tear (**a**) and stain (**b**) damages. For both the plots *x*-axis represents the values of the distances obtained and *y*-axis the number of occurrences. ${\bar{e}}_{null}$, ${\bar{e}}_{0}$ and ${\bar{e}}_{1}$ are represented by gray, green and red respectively.

Figure 10a,b represent the plot of distances ${\bar{e}}_{0}$, ${\bar{e}}_{1}$, ${\bar{e}}_{null}$ in both tests (LBP and CLBP). The plots have been cut because the populations are unbalanced and because the focus of the analysis is on the intersections of the two curves. In those plots it is possible to detect two Gaussians almost fully separated. The intersection between them (the tail of green Gaussian, delimited by orange and blue lines) represents an uncertainty zone. It is worth noting that the position of ${\bar{e}}_{1}$ in both cases (LBP and CLBP) is within this zone that confirms the meaning of distance: lower will be the distance with the nearest fingerprint and greater will be the possibility that the fingerprints are extracted from the same document. Naturally, the concept of low/great depends on the descriptor employed; in the forensics domain it is important the measure of the degree of uncertainty whenever it is available. The percentage of uncertainty zone *z* and the percentage *r* of ${\bar{e}}_{0}$ inside it gives a further degree of confidence and it is variable for each descriptor. Given a descriptor the couple (*z*,*r*) can be employed to describe the robustness of it. CLBP has the ${\bar{e}}_{0}$ range between 0.286 and 0.338 and uncertainty zone between 0.331 and 0.338 and then z=13.46%, while r=2.62% due to 22 ${\bar{e}}_{0}$ inside uncertainty zone on 837 total; LBP has z=13.56% and r=4.92%. Table 7 shows the analysis for every binary pattern tested.

Moreover, a cross-dataset analysis has been conducted to understand if there is a correlation between input and descriptor efficiency. The textures with the distance within *z* have been analyzed: CLBP has 22 distance on 837 while LBP 41 on 835. 13 are shared while others are close to *z* meaning that bad texture (in terms of acquisition) will have a bad distances (close or within *z*), independently from the descriptor.

## 6. Conclusions

In this paper, a novel approach for document identification was proposed. The method employs variants of binary pattern descriptors (e.g., LBP, LTP, SBP, CLBP) to obtain a proper fingerprint to uniquely recognize the input document, but at the same time, be easily manageable. For this reason, an additional analysis was conducted to optimize the fingerprint in terms of dimensions; it was based on PCA which has confirmed almost the same degree of confidence, reducing the fingerprint size to less than 1/100 of the original. To demonstrate the robustness of the method, the dataset was expanded by including more noisy samples, demonstrating the value of the proposed technique in real case scenarios and better results with respect to the state of the art. Finally, a further analysis on the meaning of distances was conducted, to generalize the recognition test.

## Figures and Tables

**Table 1:** VSC Settings.

Light	Longpass	Mag	Exposure	Brightness
Transmitted	VIS	2.18	Auto	60

**Table 2:** BIT Settings.

Acquisition	Exposure Time	Opening/ISO/VR	Exposure Compensation	White Balance
RAW + JPEG	1/25	F29/100/ON	−5.0	Incandescence

**Table 3:** Dataset Table.

	VSC	BIT
**CLASS**	**ACQUISITIONS**	**ACQUISITIONS**
{e,80}	2	8
{f,80}	2	8
{u,80}	2	8
{m,160}	2	-
{m,200}	2	-
DEVICE IMG	10×40=400	24×40=960

**Table 4:** Best configuration parameters and accuracy of recognition test in VSC and BIT datasets.

Dataset	Patch Size	Number of Neighbors	Radius	LBP Type	Accuracy
VSC	100	32	42	uniform	96.5%
BIT	100	24	12	default	99.2%

**Table 5:** Recognition test accuracy of the test carried out in BIT dataset employing LTP, SBP and CLBP.

	LTP	CLBP	SBP
**Accuracy**	90.83%	99.7%	99.4%

**Table 6:** Accuracy and size of CLBP fingerprints to vary of patch size.

Patch Size	Number of Bin	Accuracy	Storage Occupancy (MB)
100	4,097,000	99.7%	8
200	1,024,250	99.3%	2.2
250	655,520	97.9%	1.5
500	163,880	67.6%	0.4

**Table 7:** Percentage of uncertainty zone (*z*) and percentage of ${\bar{e}}_{0}$ inside it (*r*) for each analyzed descriptor.

Descriptor	*z*	*r*
LBP	13.93 %	4.91 %
CLBP	13.32 %	2.62 %
SBP	15.72 %	3.13 %
LTP	91.38 %	91.05 %
